# Actigraph Accelerometer-Defined Boundaries for Sedentary Behaviour and Physical Activity Intensities in 7 Year Old Children

**DOI:** 10.1371/journal.pone.0021822

**Published:** 2011-08-11

**Authors:** Richard M. Pulsford, Mario Cortina-Borja, Carly Rich, Florence-Emilie Kinnafick, Carol Dezateux, Lucy J. Griffiths

**Affiliations:** 1 College of Life and Environmental Sciences, University of Exeter, Exeter, United Kingdom; 2 Medical Research Council Centre of Epidemiology for Child Health, University College London Institute of Child Health, London, United Kingdom; 3 School of Sport and Exercise Sciences, University of Birmingham, Birmingham, United Kingdom; Pennington Biomedical Research Center, United States of America

## Abstract

**Background:**

Accurate objective assessment of sedentary and physical activity behaviours during childhood is integral to the understanding of their relation to later health outcomes, as well as to documenting the frequency and distribution of physical activity within a population.

**Purpose:**

To calibrate the Actigraph GT1M accelerometer, using energy expenditure (EE) as the criterion measure, to define thresholds for sedentary behaviour and physical activity categories suitable for use in a large scale epidemiological study in young children.

**Methods:**

Accelerometer-based assessments of physical activity (counts per minute) were calibrated against EE measures (kcal.kg^−1^.hr^−1^) obtained over a range of exercise intensities using a COSMED K4b^2^ portable metabolic unit in 53 seven-year-old children. Children performed seven activities: lying down viewing television, sitting upright playing a computer game, slow walking, brisk walking, jogging, hopscotch and basketball. Threshold count values were established to identify sedentary behaviour and light, moderate and vigorous physical activity using linear discriminant analysis (LDA) and evaluated using receiver operating characteristic (ROC) curve analysis.

**Results:**

EE was significantly associated with counts for all non-sedentary activities with the exception of jogging. Threshold values for accelerometer counts (counts.minute^−1^) were <100 for sedentary behaviour and ≤2240, ≤3840 and ≥3841 for light, moderate and vigorous physical activity respectively. The area under the ROC curves for discrimination of sedentary behaviour and vigorous activity were 0.98. Boundaries for light and moderate physical activity were less well defined (0.61 and 0.60 respectively). Sensitivity and specificity were higher for sedentary (99% and 97%) and vigorous (95% and 91%) than for light (60% and 83%) and moderate (61% and 76%) thresholds.

**Conclusion:**

The accelerometer cut points established in this study can be used to classify sedentary behaviour and to distinguish between light, moderate and vigorous physical activity in children of this age.

## Introduction

The importance of physical activity for healthy child development is well established. There is evidence that sedentary behaviour and low levels of physical activity in childhood are associated with an increased risk of childhood obesity as well as with a range of chronic adult disease risk factors including hypertension, insulin resistance and dyslipidaemia [1]. Accurate and valid assessment of sedentary behaviour and physical activity levels during childhood is therefore integral to the understanding of their relation to later health outcomes, as well as to documenting their frequency and distribution within a population.

The development of accelerometer technology has provided a robust alternative to the methods of physical activity assessment based on self report traditionally employed in large scale epidemiological studies. Cost, practicality, and a high subject burden prevent direct observation of physical activity being feasible in population based physical activity research. Self report measures of physical activity in children are of limited validity [2] and proxy report by parents can be unreliable, especially in school-aged children.

Accelerometry is an attractive option as it provides an objective measure of activity frequency, intensity and duration. It also eliminates recall and social desirability bias and may overcome the challenges posed by difficulties in language and literacy [3]. Continuing technological development with subsequent increases in battery life and decreases in unit cost have allowed accelerometers to become feasible in large scale population based physical activity studies.

The Actigraph accelerometer (Actigraph, Pensacola, Florida) has been extensively and successfully used to assess physical activity in children in both small [4], [5], [6], [7], [8], [9] and large scale [10], [11] epidemiological studies. Accelerometers provide dimensionless physical activity scores in ‘counts’ which are summarised over a user specified time period or epoch. By calibrating accelerometer counts with an objective ‘gold standard’ measure of energy expenditure (EE) such as oxygen consumption over a range of exercise intensities, threshold values for accelerometer data can be established to delineate categories of physical activity intensity. Accelerometer-based data can then be summarised according to these threshold values to determine whether, at a population level, physical activity meets current public health guidelines, which are conventionally expressed in terms of the minutes spent each day in moderate to vigorous physical activity (MVPA).

While accelerometer counts have been calibrated with respect to EE data in a number of studies of different age groups using either structured [9], [12] or free living activities [13] these studies have differed widely in design, methods and statistical approaches to data reduction and analysis, resulting in considerable variation in the threshold values published in the literature. The majority of calibration studies have focused on discriminating between differing intensities of physical activity (light, moderate, vigorous). However, accelerometers are also able to identify sedentary behaviour which is not simply the absence of physical activity. It has been suggested that sedentary behaviour comprises the majority of young children's time [14] and it is increasingly considered as an independent risk factor for a number of metabolic disorders [15] with its own patterns and determinants rather than simply one extreme of the physical activity continuum. Few studies have objectively assessed free-living sedentary behaviours in children, and those that have, have included considerably different age groups [5], [8].

The Millennium Cohort Study (MCS) is a nationally representative, UK-wide, cohort study of 12768 children born in the new century (between September 2000 and January 2002). A range of social, economic and health-related information has been collected from cohort members at home interviews held at ages nine months and three, five and seven years. At the age seven year interview, these data were enhanced by measures of physical activity obtained by accelerometer. The present study aimed to calibrate accelerometer counts against measured EE (kcal.kg^−1^.hr^−1^) in a sample of children of similar age to those participating in the MCS in order to establish thresholds which define sedentary behaviour and light, moderate and vigorous activity based on accelerometer counts, and to do this using entirely self-paced rather than structured activities since the former are more representative of free living activities of children at this age. The overall aim is to use these thresholds to summarise the physical activity data collected from the MCS, and other large epidemiological studies.

## Methods

### Participants

The study sample consisted of children aged between 7 and 8 years attending a North London primary school. Information letters were sent to the parents of all 83 children in the relevant year group inviting them to participate in this study. Written consent was obtained from the parent/guardian of 55 children prior to participation in study. This study was approved by the University College London Research Ethics Committee (reference 1325/001).

### Anthropometry

Height was measured using Leicester Height Measure Stadiometers (Seca Ltd, Birmingham, UK), recorded to the nearest 0.1 cm. Weight (to the nearest 0.1 kg), and body fat percentage (to the nearest 0.1%) were measured using an electronic body composition scale (Tanita BF 522W, Middlesex, UK). Waist circumference was measured (to the nearest 0.1 cm) using a SECA tape (SECA, Hamburg, Germany), midway between the costal margin and the iliac crest. Two measurements were taken and their mean was recorded.

### Accelerometry

Measurements were made using the Actigraph GT1M uni-axial accelerometer (Actigraph, Pensacola, Florida). This uses a piezoelectric lever to detect accelerations in the vertical plane in the range of 0.05–2 g. This range is consistent with normal human movement and allows the rejection of high intensity vibrations. Flexion of this lever caused by movement generates a signal proportional to the amount of acceleration. This signal is then summed over a user defined time period (epoch) which may range from 1–240 seconds. It is small (38 mm×37 mm×18 mm ), lightweight (925 g) and has been demonstrated to measure physical activity in children reliably when compared with heart rate monitoring [16] indirect [9] and room [4] calorimetry, and doubly labelled water [17] techniques.

While a number of previous studies have used one minute [4], [9] or 30 second epochs [8] it has been suggested that the sporadic nature of children's movements when compared to adults requires more frequent assessment [18]. In view of this, in the current study we used 15 second epochs. Participants wore the accelerometer on a flexible elastic belt worn round the waist, in the right midaxillary line and level with the iliac crest. Data were downloaded immediately following completion of the protocol using the Actigraph software version 3.8.3 (Actigraph, Pensacola, Florida).

### Indirect Calorimetry

Oxygen consumption (VO_2_) and heart rate were measured using a portable breath by breath metabolic unit developed by COSMED (Model K4b_2_, Rome). This is a small (70 mm×50 mm×100 mm), lightweight (475 g) indirect calorimetry system that is worn in a chest harness. It is ideally suited to the determination of EE in non laboratory settings, and has been demonstrated to be a valid measure of oxygen uptake in both adults [19] and children [20]. All expired gases pass through a face mask connected to a bidirectional flowmeter to O_2_ and CO_2_ analysers via a sample line, allowing air flow volumes and fractions of expired oxygen (FEO_2_) and carbon dioxide (FECO_2_) to be measured. On each day of testing the unit was warmed up for 30 minutes and a delay calibration (to account for the delay between expiration and gas analysis) was carried out according to the manufacturer's guidelines. Prior to each test the unit was calibrated using a reference gas of known volume (5.2% CO_2_, 16.0% O_2_, 78.8% N).

### Protocol

All activities were performed indoors in the school's own gymnasium. Three children took part each test day, two in the morning and one in the afternoon session. The study protocol and equipment were explained to each child, anthropometric measurements obtained and the accelerometer and COSMED devices positioned. The COSMED and accelerometers were then synchronised and the test initiated. Each participant was required to perform seven activities of increasing intensity. These activities were selected to provide a full range of physical activity intensities, from sedentary to vigorous, which also reflected free living activities typical of children of this age. The activities were as follows:

1. Lying Down – subjects lay down for 30 minutes while watching a DVD.
2. Sitting – subjects sat upright on a bench while playing a computer game for 5 minutes.
3. Slow walking – subjects were instructed top ‘walk slowly’ round a marked track for 5 minutes.
4. Brisk walking – subjects were instructed to ‘walk quickly’ round a marked track for 5 minutes.
5. Jogging – subjects were instructed to ‘jog’ round a marked track for 5 minutes.
6. Hopscotch –subjects played hopscotch at their own pace for 5 minutes.
7. Basketball – subjects performed a basketball drill involving, dribbling, running and shooting for 5 minutes.

All activities were self paced. The walking and jogging activities took place around a marked 10 m×4 m track. There was a brief interval (<2 mins) between each activity to allow for movement of equipment, although activities 2, 3 and 4 (slow walking to jogging) were performed continuously. All activities were 5 minutes in duration with the exception of the first activity (lying down) which lasted 30 minutes in order to achieve EE values close to those of resting metabolism (note these values were used to represent sedentary behaviour and not to establish basal metabolic rate). Oxygen consumption and accelerometer counts were recorded throughout.

### Data reduction

The COSMED and accelerometer data were exported and aligned using a specially designed Microsoft Access macro, and a two minute sample from each activity period selected for analysis. For all activities, lying down excepted, data were sampled between minutes 2.5 and 4.5 to ensure that participants had achieved steady state EE [21] in each activity and to minimise the effect on VO_2_ of the anticipation of the end of each task. Data were taken between minutes 22.5–24.5 for the lying down period. VO_2_ was converted to units of EE (kcal.kg^−1^.hr^−1^) using the constant 1 L O_2_ = 4.825 kcal [22]. O_2_ data were then converted into METs. The standard definition of 1 MET as being equal to a VO_2_ value of 3.5 ml.kg^−1^.min^−1^ is inappropriate for use with children as VO_2_ can decline from ∼6 ml.kg^−1^.min^−1^ at age 5 to 3.5 ml.kg^−1^.min^−1^ at age 18 [23]. Since participants were measured in a school environment and were realistically not able to attend the testing sessions in a fasted state we considered it was not feasible to attempt accurate measurement of basal metabolic rate (BMR) in this setting. Furthermore, existing published equations have been well validated. In view of this we predicted BMR in kilocalories for each child using Schofield's gender specific equations based on age, height and weight [23]. MET values for each activity were then calculated as total EE divided by individual BMR. Accelerometer counts in 15 second intervals, were summed for the first and second minutes of the sample period (i.e. using 8 observations). One mean value in counts per minute was then compared to corresponding MET values across each 2 minute sample.

### Statistical Methods

All statistical analyses were performed using R version 2.12.1 [24]. Intraclass correlations coefficients (ICC) for EE in METs and accelerometer counts per minute for each activity were calculated across the 2-minute sample period for each child, thus eight individual measurements contributed to the ICC's. Functions in the R library (psychometric) [25] were used to obtain confidence intervals for the ICC estimates and if they were not significantly different from zero it was assumed that the accelerometer counts were stable during this interval, in which case mean values were used in subsequent analyses.

The Shapiro-Wilk test was used to assess normality [26]. Pearson or Spearman correlation coefficients were used to test the association between METs and accelerometer counts, depending on whether the distribution of the corresponding activity was considered Normal. Grubbs tests [27] as implemented in the R library (outliers) [28] were used to identify and remove outliers on a one at a time basis. For skewed distributions outliers were defined using the method by Huber and Vendervieren [29]. Paired *t*-tests with Welch's correction to account for unequal variances were used to compare gender subgroups. Wilcoxon-Mann-Whitney tests were used to assess differences in the energy expenditures of each activity by time of day in which the child was assessed (morning or afternoon). Regression modelling was used to assess the proportion of the variance in accelerometer counts that could be attributed to the height of the participant.

Three cut points were established, assuming a normal distribution for counts in each activity using accelerometer data for sitting, slow walking, brisk walking and jogging only. These three cut points represent the boundaries between sedentary behaviour, light activity, moderate activity and vigorous activity. We used linear discriminant analysis (LDA) as implemented in R library (MASS) [30] to determine the two optimal bounds separating the three non-sedentary activity groups. LDA produces, for each observation, a vector of posterior probabilities belonging to each level of the known activities being performed. Ideally these individual probabilities would be very close to 1 for one particular group and close to 0 for the other possible groups. We obtained the boundaries as the count values at which the posterior probability functions for each activity intersect.

Receiver Operating Characteristic (ROC) curves, as implemented in R Library (ROCR) [31] were used to assess the discriminatory power of the cut points proposed by LDA via their sensitivity and specificity. ROC curves were calculated for values across the range of observed accelerometer counts. Values of 1-specificity and sensitivity corresponding to the cut point values on the ROC curves were plotted and compared with the optimal sensitivity and specificity achievable with a particular ROC curve. This optimal value is the point minimising the distance between the calculated ROC curve and the point representing perfect classification, i.e. complete specificity and complete sensitivity. We also obtained the area under the ROC curve (AUC) which condenses the shape of the ROC curve into one number. If AUC = ½ then the model's predictions are equivalent to a random allocation meaning that the model does not discriminate between pre-defined groups, whilst AUC = 1 implies perfect classification. For each of the activities considered we tested the null hypothesis that the AUC is ½ against the composite alternative AUC>½.using the procedure based on the Wilcoxon-Mann-Whitney *U* statistic described in Mason & Graham [32].

The performance of the cut points was also evaluated by examining the misclassification rate obtained by predicting the corresponding physical activity intensity using the fitted linear discriminant model and comparing these predictions with the observed physical activity groupings.

We used the running lines smoother implemented in the supsmu function [33] in R library(stats). This is a running lines smoother that represents the data structure in a scatterplot. The smoothness of the fitted line, expressed as the width of the window around each point considered is decided in an adaptive manner depending on the local variation of the scatterplot.

## Results

Of the 55 children who consented to take part, 53 (29 male) completed the study and were included in the analysis; of these, 39 were assessed in the morning and 14 in the afternoon. The mean sample values for height (132.9±6.5 cm), weight (31.3±6.8 kg), body mass index (17.6 kg/m^2^±2.7 kg/m^2^) and waist circumference (61.1 cm±8.2 cm) did not differ significantly by gender. Mean predicted BMR for the sample was 1.58±0.18 kcal.kg^−1^.hr^−1^ or 5.46 mlO_2_.kg^−1^.min^−1^ which is equivalent to the lower EE values recorded during the sedentary activities. ICC estimates, calculated with eight consecutive measurements obtained over the 2 minutes of observations showed no significant variation within individuals across any of the seven sample periods; they were all considerably large, ranging from 0.77 to 0.90, and in all cases the standard errors yielded tight confidence intervals; as they were all smaller than 12% of the estimated ICC. This indicates that accelerometer counts were stable and that steady state activity was achieved for all activities. Data were therefore summarised as a mean for the 2 minute sample period from each activity and used in subsequent analyses. The means of each physical activity for all subjects were therefore included in the analysis. There were no significant differences in the energy expended for a given activity by the time of day in which the child completed the protocol (Wilcoxon-Mann-Whitney tests, *p*>0.01)

As expected, accelerometer counts for the sedentary activities were not normally distributed due to the high proportion of zero counts. In 35 (66%) of children the mean accelerometer count value for the sitting activity was 0; there were three outliers (251.0, 292.5, 483.0) confirmed by the method described in Hubert and Vandervieren [29] and the rest of the values were 1.5, 3.0, 3.0, 3.0, 4.5, 5.5, 5.5, 6.0, 10.5, 11.0, 11.0, 71.5, 94.5, and 96.5. thus we decided to establish a cut-point of 100 cpm to separate sedentary from non-sedentary behaviour. The distributions of accelerometer counts for slow walking and jogging did not deviate significantly from normality (*p* = 0.53 and *p* = 0.44). However, accelerometer data for brisk walking were not normally distributed (*p*<0.001) due to two outlying values (mean values of 6459 counts.min^−1^ and 6684 counts.min^−1^ compared to the sample mean of 2879). Grubbs' test identified these as the only outliers (*p* = 0.002 in both cases) in the dataset. Therefore for the purposes of the LDA the distribution of accelerometer counts for brisk walking was considered normal; the only consequence of deviating from this assumption regarding the LDA would be a slight increase in the misclassification rate caused by these two outliers.

**Figure 1:**
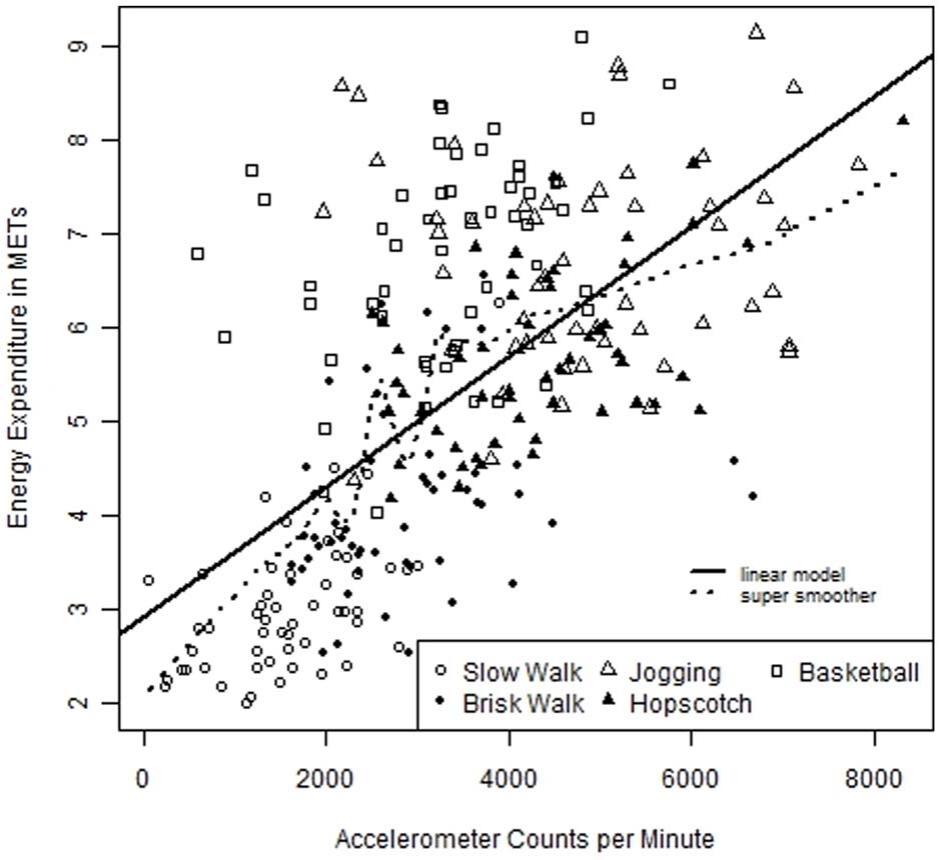
Relationship between accelerometer counts per minute and energy expenditure in METs. Data included for five of the seven activities (data for lying and sitting excluded due to the high number of zero values).

There was a significant relationship between METs and counts for all of the non-sedentary activities (*p*<0.001) with the exception of jogging (Figure 1). The mean EE (kcal.kg^−1^.hr^−1^ and in METs) and corresponding accelerometer counts (per minute) for each of the seven activities are summarised in Table 1. The accelerometer counts for the two sedentary activities are not reported in the Table because 62% and 66% of the values for lying and sitting respectively were zero.

**Table 1:** Energy expenditure (EE) in kcal.kg^−1^.hr^−1^ and METs, and accelerometer counts (per minute) for each activity.

	Activity	EE (kcal.kg^−1^.hr^−1^)	EE (METs)	Counts.min^−1^
Sedentary	Lying	2.01 (0.54)	1.26 (0.26)	62%*
	Sitting	2.25 (0.74)	1.42 (0.45)	66%*
Light	Slow walking	4.74 (1.06)	3.02 (0.75)	1592 (783)
Moderate	Brisk walking	6.50 (1.51)	4.15 (1.08)	2879 (1042)
Vigorous	Jogging	10.59 (1.58)	6.77 (1.30)	4835 (1424)
	Hopscotch	8.96 (1.31)	5.74 (1.13)	4299 (1162)
	Basketball	10.67 (1.98)	6.83 (1.51)	3301 (1079)

EE = Energy expenditure calculated from VO_2_ measures as 1LO_2_ = 4.825 kcal.

METs calculated as activity EE (kcal.kg^−1^.hr^−1^)/individual BMR.

Values expressed as mean (standard deviation).

* = % of zero counts.

The variance in accelerometer counts was not significantly attributable to the height of the participants in six of the seven activities. The only exception was the slow walking activity where height accounted for a relatively small proportion (just over 5%) of the variance (*p* = 0.04). The lowest values for counts and EE were observed during the lying and sitting activities. These activities also provided the smallest variation in METs and counts. The jogging activity yielded the highest accelerometer count values. Mean EE was highest during basketball although the mean accelerometer count value for this activity was significantly lower than those recorded for both jogging and hopscotch. This may be indicative of the inability of waist mounted accelerometers to accurately assess the upper body movements involved in this activity. The hopscotch activity yielded high count values but EE values were far lower than expected. This may be attributed to fatigue (observed in almost all subjects at this point) caused by the continuous and progressive nature of the three preceding activities (slow walking, fast walking and jogging) affecting the intensity at which this activity was performed. Due to these measurement issues and the hopscotch and basketball activities were excluded from further analyses. The relationship between EE during activity and accelerometer counts is illustrated in Figure 1. The linear model represented there was fitted using a linear mixed effects procedure with a random effect on the intercept to account for the repeated observations from each child. The dotted line corresponds to the locally adaptive super smoother function [33].

Mean (± standard deviation) distances covered during the slow walk, brisk walk and jogging activities were 278±50 m, 387±57 m and 532±85 m respectively. These distances did not differ by gender for brisk walking and jogging although boys on average walked significantly (*p*<0.001) faster and covered more distance during the slow walk task.

**Figure 2:**
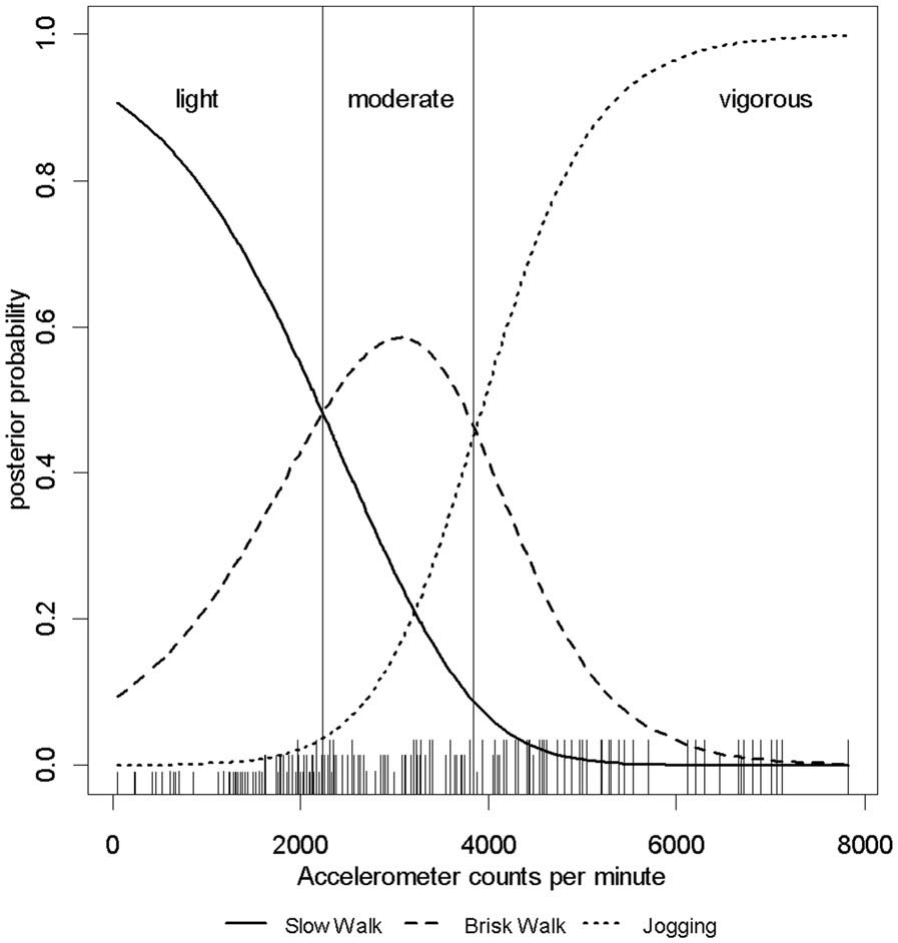
Posterior probability vectors for light, moderate and vigorous activity. Posterior probability vectors for light, moderate and vigorous activity and the cut points which occur at the points of intersection. Activities corresponding to the observed counts are indicated by the height of the ticks on the *x*-axis: the shortest ticks represent slow walking and the tallest represent jogging.

**Figure 3:**
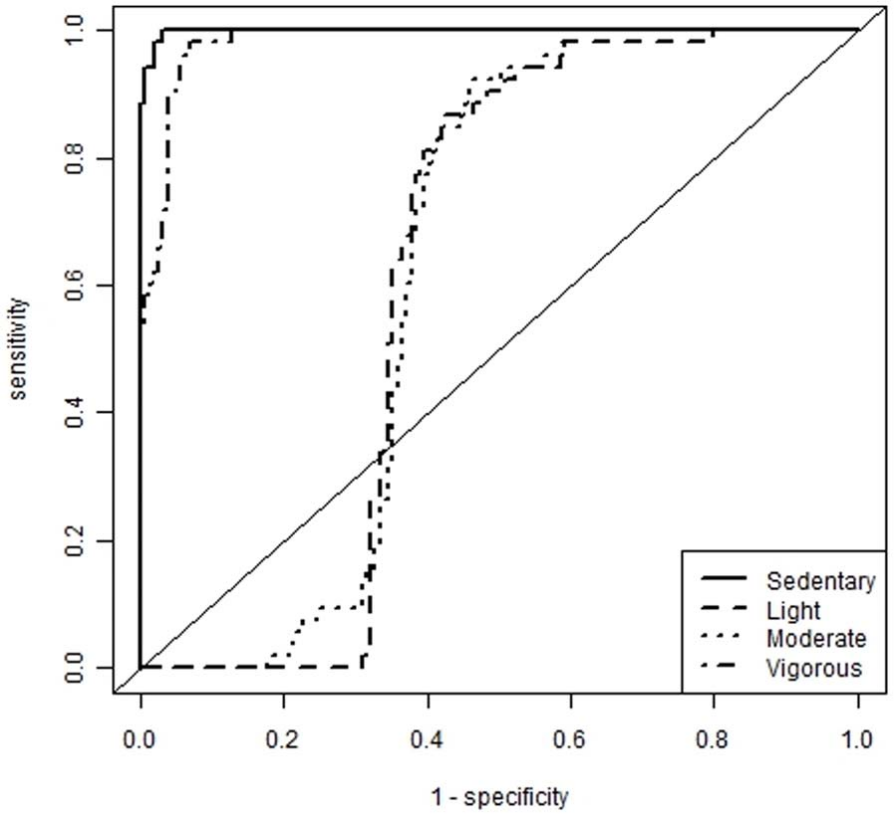
ROC curves including for sedentary, light, moderate and vigorous activity.

Figure 2 shows the posterior probability vectors for each activity and the cut points characterised as the intersections of these curves. These cut points for sedentary behaviour and for light, moderate and vigorous intensity exercise defined by the linear discriminant analysis were 100, 2240 and 3840 counts per minute and are shown in Table 2 along with the corresponding sensitivity and specificity values and the AUC obtained from the ROC curve analysis. Table 2 also describes the ROC curves which are illustrated in Figure 3. These cut points provided excellent discrimination of both sedentary behaviour and vigorous physical activity as demonstrated by AUC values of 0.98 for both activities, in contrast to light and moderate activities which were not so not as well defined by the cut points (AUC 0.61 and 0.62 respectively). The overall misclassification rate for these four cut points, calculated as the total number of correctly predicted classifications divided by *n* (53)×4, was 22.2%. The null hypothesis that all four ROC curves constructed did not predict intensity of physical activity accurately was significantly rejected (*p*≤0.003).

**Table 2:** Cut points in counts per minute (cpm) for each activity intensity category and their corresponding optimal sensitivity and specificity, and area under the ROC curve (AUC) values.

	Intervals (cpm)	Optimal sensitivity (%)	Optimal specificity (%)	Achieved sensitivity (%)	Achieved specificity (%)	AUC
Sedentary	≤100	99	97	99	97	0.98 (p<0.001)
Light	100–2240	60	83	59	83	0.61 (p<0.005)
Moderate	2241–3840	61	76	60	76	0.60 (p<0.005)
Vigorous	≥3841	95	91	95	91	0.98 (p<0.001)

## Discussion

### Summary

We have demonstrated a strong association between accelerometer counts and EE measured in a sample of seven year old children over a range of free-living activities. Using these data we have, for the first time established cut points (counts.min^−1^) to identify sedentary behaviour (≤100) and to differentiate between light (≤2240), moderate (≤3840) and vigorous (≥3841) physical activity in UK 7 year olds. Using ROC curve analysis we have demonstrated that these cut points provide good discrimination between physical activity intensity categories, especially for sedentary behaviour and vigorous physical activity, with an overall low misclassification rate and are therefore a useful tool in analysing population physical activity data for this age group.

### Comparisons with existing research

Comparison of our findings with those reported from other published calibration studies are complicated due to variation in sample age, criterion measure, accelerometers used and calibration activities employed. However the cut points defined in the current study are very similar to those previously observed by Evenson *et al.* [3] in a similar age group (sedentary ≤100 counts.min^−1^, light ≤2292 counts.min^−1^, moderate ≤4008 counts.min^−1^, vigorous ≥4009 counts.min^−1^). In Evenson *et al.'s* study two accelerometers including the Actigraph were calibrated against the COSMED K4b^2^ over 10 activities covering a range of intensities. The cut points obtained for the Actigraph were similar to those obtained in our study. AUCs reported by Evenson *et al.* were also similar to those reported in our study for sedentary behaviour (0.98), lower for vigorous physical activity (0.86), and higher for moderate physical activity (0.85). The better discrimination between moderate and light physical activity in Evenson *et al.*'s study may be due to the more structured nature of the activities used in their protocol which were in addition all performed at a predetermined pace. Locomotor activities such as walking and running were performed on a treadmill at a specified speed, while activities such as stair climbing and ‘jumping jacks’ were performed in time with a metronome. A number of studies have used similar pacing techniques [4], [12], [34], [35]. This uniformity of activity would ensure significantly reduced inter individual variation in EE and accelerometer counts when compared to the current study in which all activities were entirely self paced. The focus in the current study was to calibrate the accelerometer for use in the measurement of free-living activities. It could be argued that controlling the pace and intensity of activities, such as walking and running, does not accurately reflect natural activity for all individuals. However, Pate *et al.* [12] cross validated Actigraph cut points established for MVPA and VPA in 3–5 year old children using structured activities with periods of unstructured play involving no prescribed activities and found good agreement between the two.

Mattocks *et al.* [36] established cut points for a range of MET values using self paced activities similar to those used in the current study but for an older age group (mean age 12.4 years). Their derived cut points were considerably higher than those established in the current study (moderate ≥3581 counts.min^−1^, vigorous ≥6130 counts.min^−1^). This may reflect differences in the approach to estimation of BMR which is needed to determine EE. Resting metabolic rate has been measured under controlled, fasted conditions [3], [8] using direct calorimetry [35] in a number of studies but was not considered feasible in our study which took place during school hours. Hence in our study we calculated EE in METs using individual BMR values predicted from previously validated age specific equations using height and weight [23]. In contrast, Mattocks *et al.* [36] used the mean lowest VO_2_ value recorded during their five minute sedentary activity period. Although Mattocks *et al.* stipulated a one hour fast prior to their assessment it is unclear whether this is sufficient to ensure a true measure of basal metabolic rate. Overestimation of BMR would tend to produce systematically higher cut off points and may account in part for the higher values reported by Mattocks *et al.* in comparison with the current study.

Evenson *et al.* [3] derived cut points for two age groups (5–6 and 7–8 years) and concluded, having compared the respective ROC curve analyses, that no significant differences existed between the sets of cut points and that age-specific intensity category boundaries were not needed in this age range. In contrast, other authors have argued that age-specific cut points are needed [7]. Puyau *et al.* [4] reported considerably higher cut points than in the current study (sedentary ≤800 counts.min^−1^, light ≤3200 counts.min^−1^, moderate ≤8200 counts.min^−1^, vigorous ≥8201 counts.min^−1^); this may be attributable to the sample which included children with a considerably greater age range (6–16 years). It has been postulated that variation in height, leg length, and movement economy with age may affect count values registered by a hip mounted accelerometer [7]. The wide ranging cut points reported for the various age groups examined in the literature could also be seen to support this argument. Stone *et al.* [37] investigated the effect of leg length as well as age on the accuracy of accelerometer based EE prediction equations, and found that both factors influenced predicted values. When considering the objective measurement of population level physical activity, using accelerometer thresholds based purely on physical characteristics would require a potentially infinite number of cut points and prohibitively complicate data collection. In addition, despite the range of heights in the current sample (120.2 cm–147.4 cm), height was only seen to explain a small portion of the variation in accelerometer counts for one of the seven activities. The variation in cut points in the existing literature may be a reflection of behavioural differences in sedentary and physical activities between the age groups. Physical activity changes as a child develops, moving from sporadic informal play in early childhood to activities that begin to mirror those of adults in adolescence. When activities are self paced children of different age groups may approach what could be broadly described as the same activity or game in very different ways and with very different movement patterns depending on their own experience. That accelerometers have been shown to have more or less difficulty accurately capturing certain activities makes the way these activities are typically performed an even more central issue. The age of the individual being assessed therefore becomes of the utmost importance. We are therefore confident that the Actigraph cut points established here can be used to evaluate time spent engaged in sedentary behaviour and light, moderate and vigorous activity in children of this age group.

A number of studies have used linear regression analysis to obtain cut points [4], [6], [12], [35]. This method is limited by its assumption that a linear relationship exists between EE and accelerometer counts; this is not always the case [38]. The slight plateaux in accelerometer counts (per minute) with continuing increase in EE appears in the fitted super smoother shown in Figure 1. This may indicate an inability of accelerometers to define physical activity accurately at high levels of EE or an anaerobic contribution to exercise metabolism which causes the relationship to become more complex [3]. In the current study LDA was used to establish boundaries for known subgroups (physical activity categories) present in the data. LDA is the natural technique to use to construct boundaries when the subpopulations (in this case physical activities) to be identified are known; this is in contrast to a situation in which they are latent or unobserved, in which finite mixture regression models [39] would be adequate. Few studies have evaluated accelerometer cut points based on the optimal sensitivity and specificity values obtained from ROC curve analyses. This method should reduce the misclassification of physical activity attributed to the wide variation in accelerometer output for a given activity [40].

### Strengths and limitations

We are confident that the experimental and analytical methodologies employed in the current study have enabled us to define robust cut points with which to identify sedentary behaviour and light, moderate and vigorous physical activity in 7 year old children. The use of a full range of age appropriate, self paced activities analysed at 15 second intervals will allow sensitive and effective analysis of everyday physical activity in children of this age using data from the MCS. With continuing advances in accelerometer technology it will be possible to measure free living physical activity over a number of days using even shorter epochs. It would therefore be useful to validate the cut points established in the current study using an even more sensitive measure of children's physical activity.

A number of limitations must be acknowledged in the current study. Data collection took place around the children's normal school day and it was therefore not possible to test each subject at the same time of day. Some children therefore took part in the activities directly after lunch which could potentially have influenced their metabolic rate and lead to the misclassification of light activity as moderate. However, Wilcoxon-Mann Whitney tests revealed that EE did not differ significantly depending with the time of day the protocol was completed. The age of the participants and the constrains of the school day also meant that obtaining basal metabolic measures under controlled and fasted conditions prior to each trial was not feasible. Therefore, values for BMR used to calculate MET values for each activity were predicted rather than being measured using a controlled protocol. However, the predicted mean BMR of 1.58 kcal.kg^−1^.hr^−1^ or 5.46 mlO_2_.kg^−1^.hr^−1^ falls well within the expected range.

In the current study activities were chosen which provided a range of intensities and reflected free living activities typical of children of the sample age. Previous investigations have used up to 10 activities [4], [6], [8], [12] however, practical considerations prevented this in the current study. Activities involving climbing and upper body movement which may be considered equally typical of this age group were not included due to the documented inability of accelerometers to accurately define external and load bearing work as well as topographical transition (i.e. lifting or walking on a slope) [41]. However, although studies such as this are limited by the capabilities of the accelerometer it has been previously observed that children's activity is largely comprised of locomotor activities [42].

In our study, the relationship between EE and counts was significant for all non-sedentary activities with the exception of jogging. It has been previously observed that accelerometer counts do not increase linearly at high speeds [43] which may account for this. A number of studies [3], [36] allowed several minutes between activities to allow VO_2_ to return close to resting values. In the current study, although a few minutes were needed to move between activities these breaks were not consistent across subjects. In addition, the two walking activities and jogging were performed continuously. This may have affected the subsequent two activities. Both counts and EE for hopscotch were significantly lower than during jogging, which may suggest an effect of fatigue from the previous 15 minutes continuous activity (slow walking, brisk walking and jogging). The basketball activity elicited the highest mean energy expenditure, but a mean counts value lower than those for either jogging or hopscotch. This indicates an inability of waist worn accelerometers to accurately determine upper body and load bearing activity [41]. The relatively low counts value for basketball compared to jogging also indicates that changes in EE may not accurately reflect changes in body movement. This would be particularly apparent in intermittent, game-type activities where body movement occurs in sporadic bursts. Treuth *et al.* [8] observed a similar effect when examining the relationship between Actigraph counts and EE during basketball. Energy expenditure would also remain high in post-exercise periods which may result in the underestimation of total EE by accelerometers [35]. Hopscotch and basketball activities were a useful inclusion as they reflect the varied nature of activities typical of this age and both demonstrated a significant relationship between EE and counts (*p*<0.001 for both). However, the jogging activity provided an adequate representation of vigorous steady state activity so data from the hopscotch and basketball activities were excluded from the linear discriminant and ROC curve analyses.

It would be useful to cross validate our findings with a larger sample of 7 year old children both under free living conditions and using controlled prescribed activities. This was beyond the scope of the current study. However, a number of studies using children of different ages have included a cross validation of cut points in their protocol [12], [34] and found good agreement between structured and free living activities.

Discussion within the published literature as to the optimal way of objectively classifying population level physical activity data is ongoing. Aside from the derivation of accelerometer cut points, pattern recognition based approaches have emerged as an alternative method of broadly classifying specific activity types to estimate EE from accelerometer data. Accelerometer data can be classified as belonging to a particular activity type by comparison with pre-determined data patterns for specific activities. Such studies have shown reasonable success in classifying a small range of controlled physical activities in adults [44], [45]. However, only two studies have attempted to apply these approaches to accelerometer data from children [46], [47] and these focussed purely on differentiating between specific activity types rather than activity intensity. Activity misclassification was also considerably higher than in previous adult studies, potentially due to the wider variation and sporadic nature of children's activity patterns compared to adults. It must also be recognised that the utility of the pattern recognition approach to free living physical activity data from population based studies would be dependent on the development of patterns from a huge range of specific activities. For this reason, for the time being the use of accelerometers cut points remain the most important tools in the surveillance of population level physical activity levels in children.

### Recommendations

The variation in cut point values derived for different age groups may be partially attributable to the different methodologies used. However, differences in the behavioural aspects of self paced sedentary and physical activities at different age groups may also have a significant effect on their measurement. Further investigation is needed into the measurement of true free living activities and the variations that could potentially exist between different age groups of UK children. Differences in the movement patterns that make up spontaneous locomotor and game type activities could certainly alter the evident relationship between EE and accelerometer counts. A better understanding of these differences may allow more effective measurement and reduce the misclassification of physical activity during objective measurement.

### Conclusion

In conclusion, the Actigraph GT1M accelerometer can be used to identify sedentary behaviour and to discriminate between light, moderate and vigorous activity in 7 year old children. The cut points defined in the current study will be useful in interpreting physical activity data from the MCS, as well as other studies examining sedentary behaviours physical activity in children of this age.
